# Electrochemical and electron microscopic characterization of Super-P based cathodes for Li–O_2_ batteries

**DOI:** 10.3762/bjnano.4.74

**Published:** 2013-10-18

**Authors:** Mario Marinaro, Santhana K Eswara Moorthy, Jörg Bernhard, Ludwig Jörissen, Margret Wohlfahrt-Mehrens, Ute Kaiser

**Affiliations:** 1 Zentrum für Sonnenenergie- und Wasserstoff-Forschung Baden-Württemberg, 89081 Ulm, Germany; 2 Electron Microscopy Group of Materials Science, University of Ulm, Albert-Einstein-Allee 11, 89081 Ulm, Germany

**Keywords:** aprotic electrolyte, impedance spectroscopy, Li–O_2_ batteries, scanning electron microscopy

## Abstract

Aprotic rechargeable Li–O_2_ batteries are currently receiving considerable interest because they can possibly offer significantly higher energy densities than conventional Li-ion batteries. The electrochemical behavior of Li–O_2_ batteries containing bis(trifluoromethane)sulfonimide lithium salt (LiTFSI)/tetraglyme electrolyte were investigated by galvanostatic cycling and electrochemical impedance spectroscopy measurements. Ex-situ X-ray diffraction and scanning electron microscopy were used to evaluate the formation/dissolution of Li_2_O_2_ particles at the cathode side during the operation of Li–O_2_ cells.

## Introduction

The development of new types of electrochemical power sources is nowadays considered a key factor for further development of hybrid and fully electric vehicles. Indeed one of the major concerns for the practical use of fully electric vehicles is the limited mileage of such vehicles. Aprotic rechargeable Li–O_2_ batteries may overcome this limitation since they can provide a much higher energy density than common Li-ion batteries. However, research about this new battery technology is still at an early stage. There are indeed still many open questions that need to be answered before proceeding for further development.

One of the main challenges is represented by the choice of a suitable electrolyte, which allows for the formation of the desired products during the operation of a typical Li–O_2_ battery. In this context, recently published literature [1–3] gives new insights about the mechanism through which the reduction and the oxidation of oxygen occur in aprotic environments. During discharge, the oxygen reduction reaction (ORR) proceeds in a stepwise fashion leading to the formation of LiO_2_ and Li_2_O_2_ as shown in the chemical reactions below. Conversely, upon charging, the oxygen evolution reaction (OER) gives O_2_ and Li^+^ back via a 2-electrons reaction.


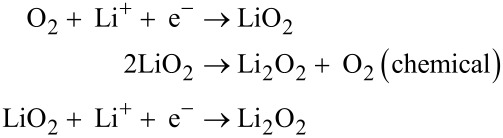


The unsuitability of commonly used electrolytes for Li-ion batteries (e.g., electrolytes based on carbonates) in Li–O_2_ cells has been demonstrated by several research groups [4–8]. Indeed, the main discharge product when using carbonates-based electrolytes is represented by the byproduct Li_2_CO_3_ rather than the desired Li_2_O_2_. On the other hand, ether-based electrolytes seem able to give the desired discharge products, even if their long-term stability is still questionable [9–15].

In view of this, we present an investigation of the lithium-oxide phases that are generated during the operation of Li–O_2_ batteries, which use LiTFSI/tetraglyme as the electrolyte. The electrochemical behaviors of the batteries were investigated by galvanostatic cycling and electrochemical impedance spectroscopy. The physico–chemical investigation of the lithium-oxide phases that form and dissolve at the cathode side upon discharge and charge of Li–O_2_ batteries has been carried out by using X-ray diffraction and SEM measurements.

## Experimental

### Electrolyte preparation

Tetraethylene glycol dimethyl ether (tetraglyme) purchased from Aldrich was dried over molecular sieves and under vacuum (at 80 °C) before being stored in an Ar-filled glovebox (MBrown), in which the levels of O_2_ and H_2_O were kept constantly below 0.1 ppm. Bis(trifluoromethane)sulfonimide lithium salt (LiTFSI) was also purchased from Aldrich and vaccum-dried before being used. The 1 M LiTFSI/tetraglyme electrolyte was prepared and stored in a glovebox.

### Electrodes manufacturing

Carbon cathodes for Li–O_2_ cells were prepared by airbrushing a suspension of Super-P (Timcal) and polyvinylidene fluoride (PvdF) in *N*-methyl-2-pyrrolidone (mass ratio Super-P/PvdF 8:2) on a gas diffusion layer (Toray paper). The obtained electrodes of 12 mm in diameter were first dried at 100 °C in order to allow the evaporation of the solvent and then further dried at 130 °C under vacuum, thus minimizing the moisture content. The average loading for all the electrodes was about 1 mg·cm^−2^ based on the carbon content.

### Electrochemical measurements

Electrochemical measurements were carried out using commercially available 3-electrode ECC–air cells (EL-cell GmbH, Germany) equipped with inlet and outlet for O_2_ purging. Lithium disks acted as the counter and the reference electrode, 1 M LiTFSI/tetraglyme and Whatman glass fiber were the electrolyte and the separator, respectively, and the Super-P based electrode was set as the working electrode. The cells were assembled in an Ar-filled glovebox (MBraun) and sealed. The batteries were then taken outside the glovebox and pure O_2_ was purged for 60 min before starting the electrochemical measurements. Galvanostatic cycles of the Li–O_2_ cells have been collected at a current of 50 mA·(g carbon)^−1^. Electrochemical impedance spectroscopy (EIS) measurements have been carried out in the frequency range between 200 kHz and 5 mHz superimposing a sinusoidal potential oscillation of ±2.5 mV. Electrochemical measurements have been carried out using a vmp 2/z (Bio-Logic, France). All potentials reported hereafter are given versus the Li^+^/Li semi-couple.

### X-ray measurements

X-ray diffraction patterns have been collected on a Siemens D5000 diffractometer equipped with a Cu Kα source and θ/2θ Bragg–Brentano geometry. For ex-situ measurements, cells were disassembled in an Ar-filled glovebox. Electrodes were first washed with tetraglyme and then vacuum dried. Finally the carbon cathodes were placed in an air-tight sample holder prior to run the measurements.

### Scanning electron microscopy

A Zeiss dual-beam NVISION 40 was used for scanning electron microscopy. The operating voltage for imaging was 5 kV. The images were acquired using a secondary-electron detector with an in-lens configuration.

## Results and Discussion

**Figure 1:**
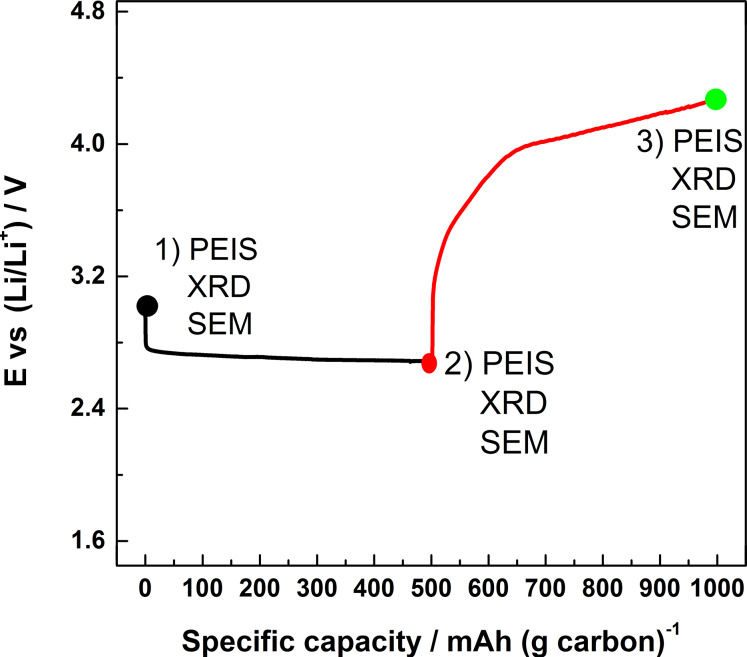
First galvanostatic discharge/charge curve of a typical Li–O_2_ battery consisting of a carbon-based cathode, lithium metal anode and LiTFSI/tetraglyme as electrolyte.

The first galvanostatic discharge/charge curve of a typical Li–O_2_ battery that has a carbon-based cathode, a lithium metal anode and LiTFSI/tetraglyme electrolyte is reported in Figure 1. The cell was cycled following a time-limited constant-current protocol. A current of 50 mA·(g carbon)^−1^ was applied for 10 h leading to a final specific capacity of 500 mAh·(g carbon)^−1^. At the expense of some energy density, the use of such protocol ensures good cyclability (more than 30 charge–discharge cycles) of the Li–O_2_ cells [10,15]. The shape of the galvanostatic curve is characterized by a flat discharge plateau at ≈2.7 V, whereas upon charging the potential of the cell rapidly increases to 3.2 V, then proceeding up to 3.9 V in a sloped manner and finally approaching the end of the charge at ≈4.3 V.

**Figure 2:**
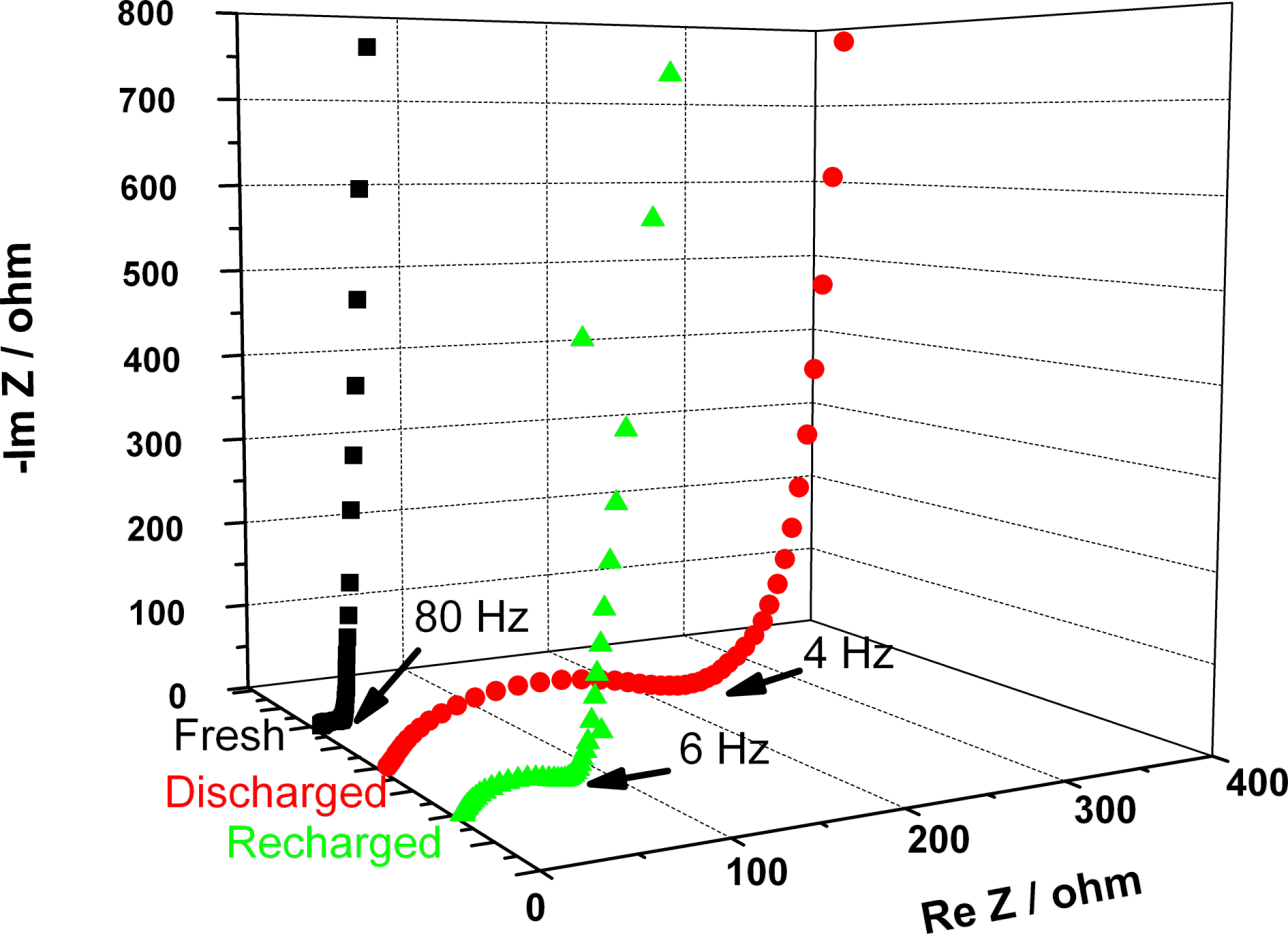
Electrochemical impedance spectra of pristine (black), once discharged (red) and re-charged (green) electrodes.

**Figure 3:**
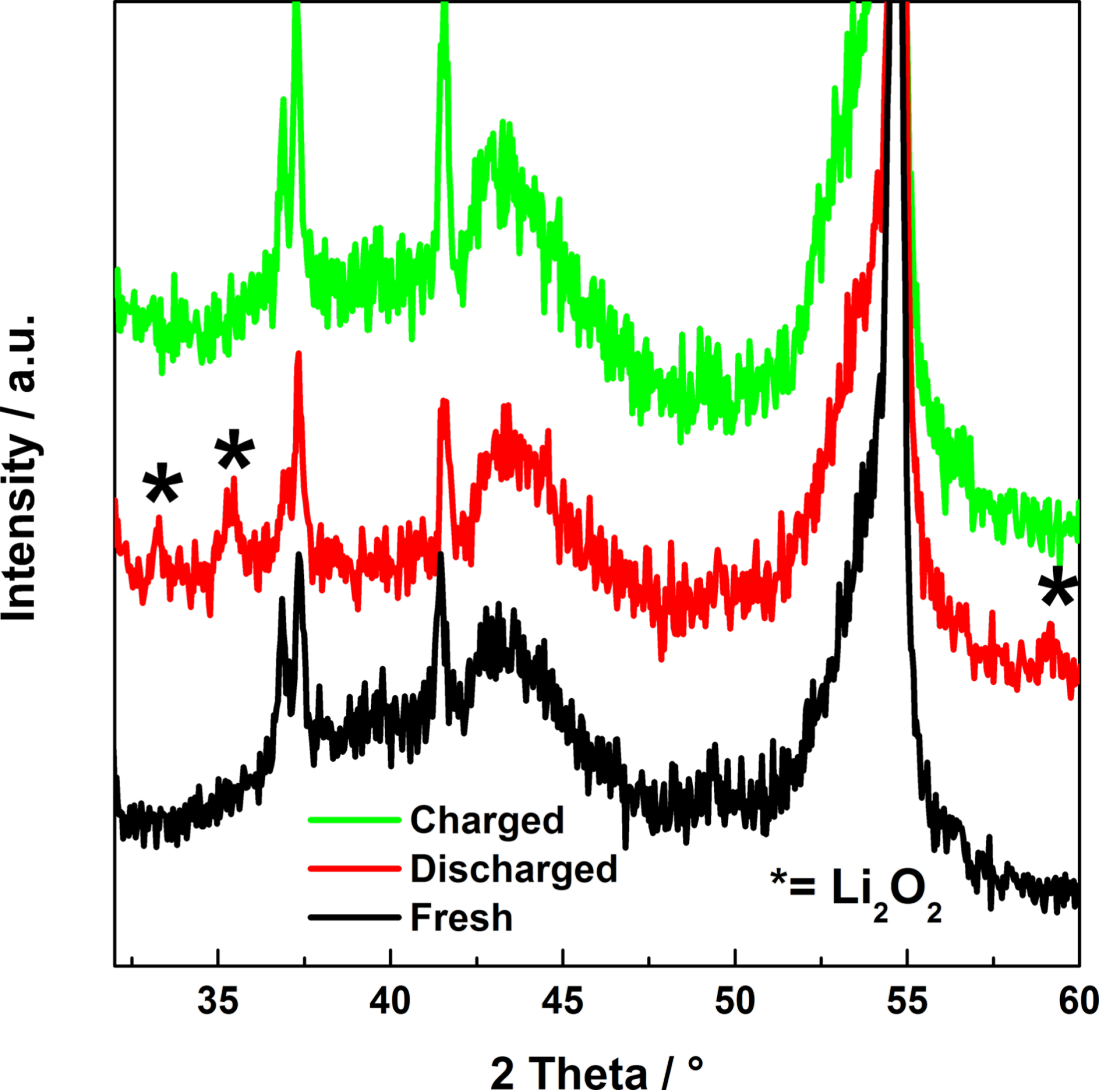
X-ray diffractograms of pristine, discharged and charged carbon cathodes. Note the additional peaks of Li_2_O_2_ (marked by asterisk) for the discharged state of the cathode.

In order to investigate the cathode/electrolyte interface, we examined the evolution of the cell impedance during the first cycle. Electrochemical impedance spectra have therefore been collected from fresh, once discharged and re-charged electrodes, as shown in Figure 2. The EIS results can be used to evaluate the formation of an insulating phase at the electrode. The 3-electrode configuration of the used cell allowed for isolating the only contribution of the cathode to the total impedance of the cell. The Nyquist dispersion of the fresh cathode reported in Figure 2 (black squares) displays a depressed semicircle from high to middle frequencies, which can be ascribed to the charge transfer resistance [16]. The impedance spectrum acquired at the end of the discharge (Figure 2, red circles) clearly shows an increased amplitude of the semicircle associated with an increased charge-transfer resistance. The observed behavior can be explained on the basis of a growing insulating phase at the cathode side and more specifically directly related to the formation of Li_2_O_2_ upon discharge, as demonstrated by the XRD results that will be discussed later on. After charging (Figure 2, green triangles) the impedance associated with the charge transfer decreases, which suggests that the formation/dissolution of Li_2_O_2_ is reversible in the system. The reasons for the different values of the charge-transfer resistances of the fresh and of the charged electrodes are still unclear. Although the XRD results (see Figure 3) do not show evidences of Li_2_CO_3_, we cannot exclude that traces of this compound, either in an amorphous state and/or below the detection limit of the X-ray diffraction technique, forms during discharge as a consequence of the instability of the carbon-based electrode [17–18] or of the electrolyte [15] during cycling. The formation of the insulating Li_2_CO_3_ within the cathode structure would explain the different charge-transfer values found for the fresh and re-charged electrodes, respectively.

By means of X-ray diffraction we confirmed the reversible formation and dissolution of Li_2_O_2_. The comparison of the diffraction patterns of pristine, discharged and recharged electrodes is reported in Figure 3. The two X-ray patterns of the fresh and of the charged electrodes show the same peaks. In contrast, the diffractogram of the electrode in the discharged state shows three additional peaks, marked with asterisks, which belong to the crystal structure of Li_2_O_2_. Apart from the Li_2_O_2_ phase we could not identify any other peaks (such as those of Li_2_O, Li_2_CO_3_ or LiF).

**Figure 4:**
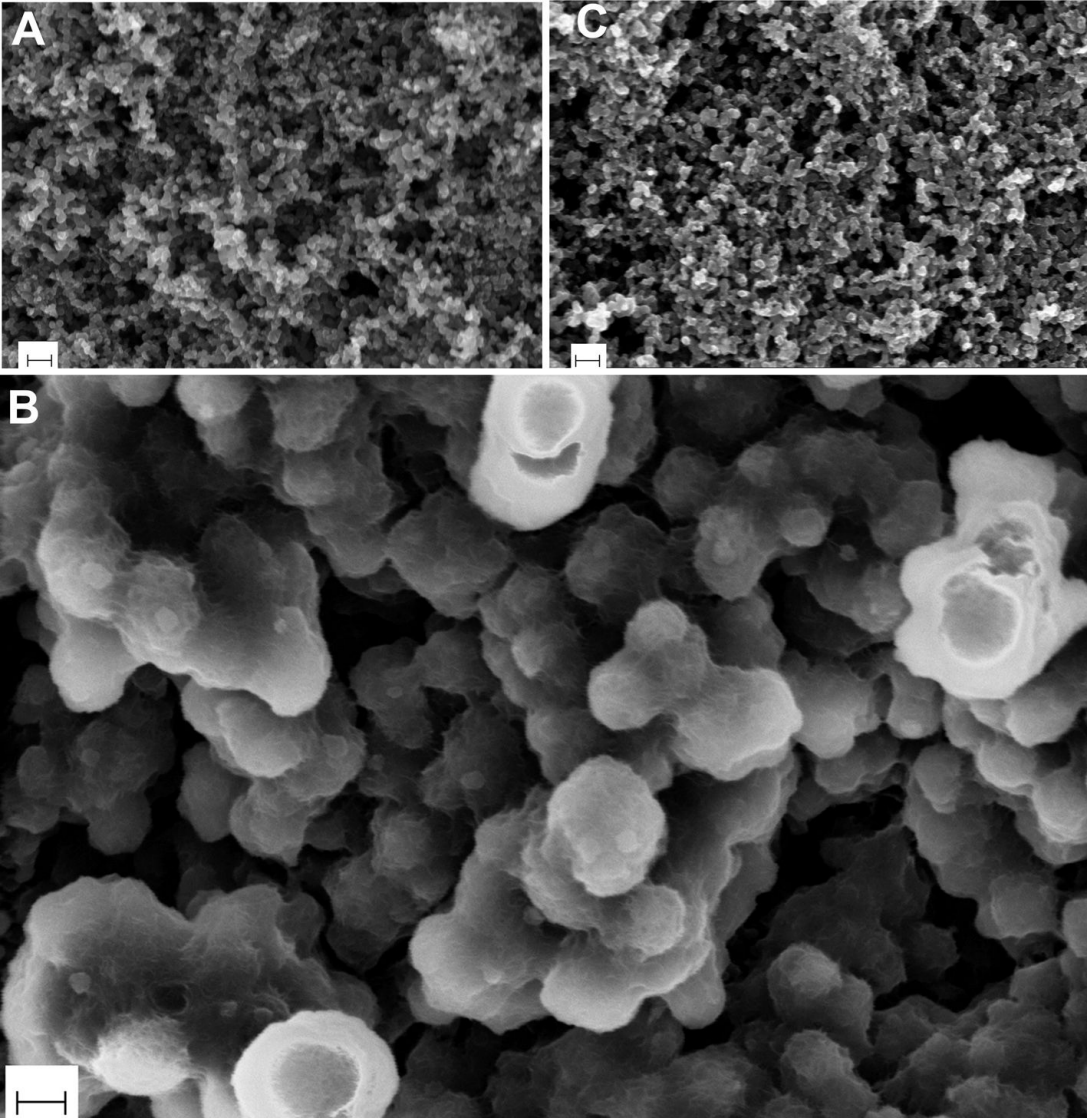
SEM micrographs of (A) pristine electrode, (B) discharged electrode for which the capacity was limited at 500 mAh·(g carbon)^−1^ and (C) recharged electrode of Li–O_2_ batteries. Note that the large Li_2_O_2_ particles in (B) appear to have a hollow structure with a smooth surface and nodular morphology. The scale bars correspond to 200 nm.

The SEM images of the electrode at pristine, discharged up to 500 mAh·(g carbon)^−1^ and recharged states are shown in Figure 4. Discharging the battery forms lithium peroxide on the cathode as seen in Figure 4B. It can be noticed that the Li_2_O_2_ particles appear to have a hollow structure with a smooth surface and nodular morphology. The dimensions of the particles are typically in the range of 200 to 350 nm. From Figure 4C it is obvious that upon recharging the battery the Li_2_O_2_ particles disappear in accordance with the expectation. Our SEM results are consistent with the XRD results shown in Figure 3, which show Li_2_O_2_ peaks in discharged case but not in the recharged case. These results are also consistent with the results from electrochemical impedance spectroscopy.

**Figure 5:**
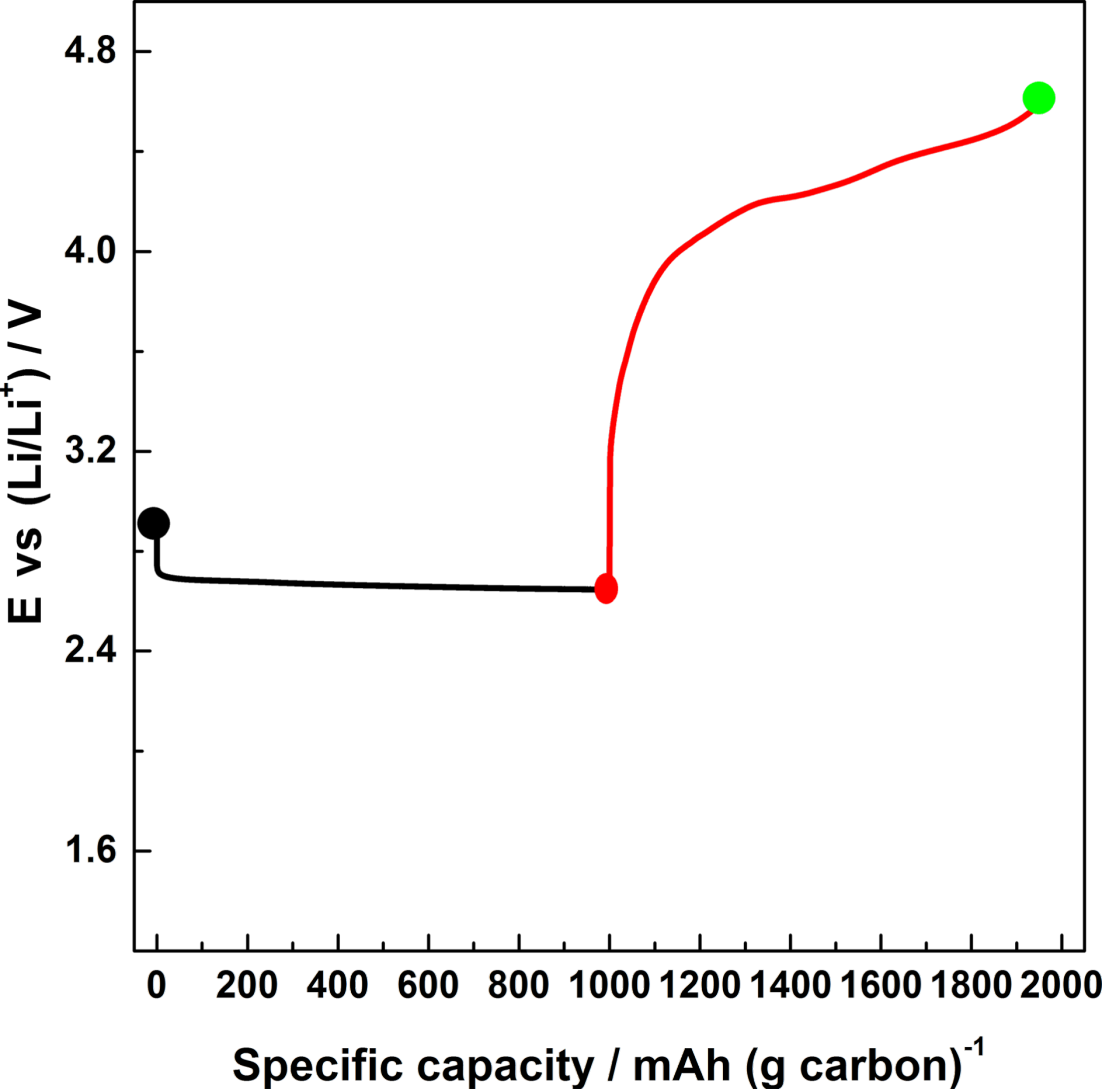
First galvanostatic curve of a Li–O_2_ battery discharged up to 1000 mAh·(g carbon)^−1^.

In order to comprehend the electrochemical and microstructural changes that occur when the depth of discharge of a Li–O_2_ battery is further increased, Li–O_2_ cells were cycled under a fixed capacity regime of 1000 mAh·(g carbon)^−1^. The galvanostatic curve referred to the first cycle is shown in Figure 5. It can be seen that the general shape of this curve is similar to that of Figure 1 even though in the latter case the capacity was limited to 500 mAh·(g carbon)^−1^.

**Figure 6:**
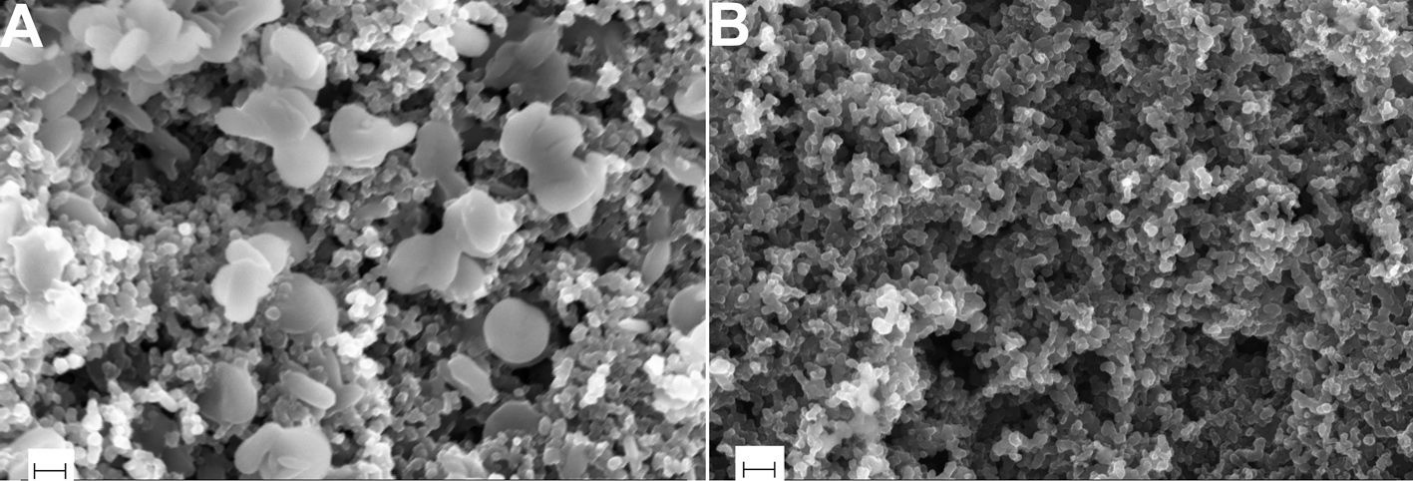
Microstructures of (A) discharged and (B) recharged electrodes. The formation of lithium peroxide crystals on the discharged electrode up to 1000 mAh·(g carbon)^−1^ is clearly visible in (A). The scale bars correspond to 200 nm.

The corresponding SEM images of the discharged and recharged electrodes are shown in Figure 6. The formation and dissolution of lithium peroxide crystals upon discharging and recharging are evident from these images. By comparing Figure 6A with Figure 4B, the following three features are obvious: (a) the morphology of the particles is rather smooth in both the cases (b) the particles in Figure 6A are filled (not hollow) in contrast to the case of Figure 4B and (c) the size of the Li_2_O_2_ particles formed in the case of Figure 6A are in the range of 250–350 nm, which is somewhat larger than in the case of Figure 4B. From point (b) we can infer that the formation mechanism of the Li_2_O_2_ must involve a transformation from a hollow to filled structure with the progression of the discharge. Moreover, the continuous coverage of Li_2_O_2_ on carbon seen in Figure 4B is starkly different from the discontinuous coverage shown in Figure 6A. This may be due to thermodynamic and kinetic factors at play during discharge which determines the overall morphology of the reaction products. Although the exact mechanism is unclear, it also explains the aforementioned transformation from hollow to filled structures. Finally, from points (b) and (c) together we can also qualitatively understand the excess mass deposited on the electrode due to a continued discharge up to 1000 mAh·(g carbon)^−1^, as would be expected.

## Conclusion

In conclusion, we demonstrated the reversibility of the oxygen electrochemical redox reaction during the operation of a Li–O_2_ battery. The use of the LiTFSI/tetraglyme electrolyte allows for obtaining the desired discharge product that is identified as Li_2_O_2_. The combination of electrochemical techniques and ex-situ analysis, such as XRD and SEM, led us to ascribe the discharge plateau to the electrochemical reduction of O_2_ which is subsequently re-oxidized upon charge. From the SEM images, it can be seen that with the progression of the discharge reaction, a hollow shell structure of Li_2_O_2_ particles forms initially which then transforms to a completely filled solid structure suggesting that the deposition mechanism must be responsible for this transformation.
